# Identifying task-relevant spectral signatures of perceptual categorization in the human cortex

**DOI:** 10.1038/s41598-020-64243-6

**Published:** 2020-05-12

**Authors:** Ilya Kuzovkin, Juan R. Vidal, Marcela Perrone-Bertolotti, Philippe Kahane, Sylvain Rheims, Jaan Aru, Jean-Philippe Lachaux, Raul Vicente

**Affiliations:** 1 Computational Neuroscience Lab, Institute of Computer Science, University of Tartu, Tartu, Estonia; 2 UMRS 449, Université Catholique de Lyon/Ecole Pratique des Hautes Etudes, 10 Place des Archives, 69002 Lyon, France; 3 University Grenoble Alpes, University Savoie Mont Blanc, CNRS, LPNC, 38000 Grenoble, France; 4 Inserm, U1216, F-38000 Grenoble, France; 5 Neurology Department, CHU de Grenoble, Hôpital Michallon, F-38000 Grenoble, France; 6 INSERM U1028, CNRS UMR5292, Lyon Neuroscience Research Center, Lyon, France; 7 Department of Functional Neurology and Epileptology, Hospices Civils de Lyon and Université Lyon, Lyon, France; 8 Institute of Biology, Humboldt University Berlin, Berlin, Germany; 9 Université Claude Bernard, Lyon, France

**Keywords:** Neural decoding, Object vision

## Abstract

Human brain has developed mechanisms to efficiently decode sensory information according to perceptual categories of high prevalence in the environment, such as faces, symbols, objects. Neural activity produced within localized brain networks has been associated with the process that integrates both sensory bottom-up and cognitive top-down information processing. Yet, how specifically the different types and components of neural responses reflect the local networks’ selectivity for categorical information processing is still unknown. In this work we train Random Forest classification models to decode eight perceptual categories from broad spectrum of human intracranial signals (4–150 Hz, 100 subjects) obtained during a visual perception task. We then analyze which of the spectral features the algorithm deemed relevant to the perceptual decoding and gain the insights into which parts of the recorded activity are actually characteristic of the visual categorization process in the human brain. We show that network selectivity for a single or multiple categories in sensory and non-sensory cortices is related to specific patterns of power increases and decreases in both low (4–50 Hz) and high (50–150 Hz) frequency bands. By focusing on task-relevant neural activity and separating it into dissociated anatomical and spectrotemporal groups we uncover spectral signatures that characterize neural mechanisms of visual category perception in human brain that have not yet been reported in the literature.

## Introduction

Our capacity to categorize sensory information allows us to quickly process and recognize complex elements in our environment. Early studies revealed strong relations between the brain activity within certain localized networks and the neural representations of certain stimulus categories, as for example faces, bodies, houses, cars, objects and words^1–7^. These early assessments also revealed brain networks’ capability to rapidly extract categorical information from short exposure to natural scenes^8–10^ based on models of parallel processing across neural networks^3,11^. In both animal and human studies, visual cortices and particularly inferior temporal cortex (ITC) appears as a key region to integrate information at the object-level^12–14^. In humans, a great deal of observations of cortical response selectivity have been achieved using fMRI, but measuring direct neuronal activity^15,16^ also revealed similar patterns. To further understand how stimulus features and perceptual experience is processed in neural networks, brain activity, especially in sensory cortices, has been decoded using a variety of methods and signals^17–19^. This decoding often relies on machine learning to avoid a priori selection of partial aspects of the data by the human observer, and unless additional analysis is performed on the model itself it does not emphasize the mechanisms of neuronal communication within and between neural networks involved in this processing.

A pervasive feature of electrophysiological neural activity are its spectral fingerprints. Neural oscillations have been proposed to reflect functional communication processes between neural networks^20–23^. Certain frequency bands are selectively associated with the operating of different cognitive processes in the human and animal brain^24–29^, and lately, direct recordings from the human cortex have revealed the remarkable representation selectivity of broadband high-gamma activity (50–150 Hz)^30–32^. Human intracranial recordings have previously shown evidence of functional processing of neural networks related to perceptual category representation^33^ and lately the prominence of broadband high-gamma activity in selective category responses in visual areas^34–38^. Yet, very little is known about the specific relation between the different components of the full power-spectrum, including high-gamma activity, and their level of selectivity in processing perceptual categories. Previous works have shown where and when perceptual category information can be decoded from the human brain, the approach introduced in this work adds to that line of research by allowing to identify spectrotemporal patterns that contribute to category decoding without the need to formulate a priori hypothesis on which spectrotemporal regions of interest are worth investigating.

In this work we capitalize on an extensive dataset of deep intracranial electrical recordings on 100 human subjects to decode neural activity produced by 8 different stimulus categories. We analyzed the decoding models built by a random forest classifier to disentangle the most informative components of the time-frequency spectrum related to the simultaneous classification of 8 different perceptual categories. Via *feature importance* analysis we quantified the contribution of each TF component into the decoding decision, which allowed us to identify the activity patterns that were either characteristic of the processing of a specific visual category or were shared by several categories. In addition to feature importance we analyzed the predictive power of each activity pattern and identified how informative was their spectral signature for the classification of visual categories. We tested the predictive power of broadband high-gamma activity in comparison to lower frequency activity as they reflect different communication mechanisms elicited by networks seemingly involved in distinct temporal windows of functional neuronal processing. Through the analysis of feature importance we show the specific neuronal spectral fingerprints from highly distributed human cortical networks that were elicited during automatic perceptual categorization. The uncovered spectral signatures provide insight into neural mechanisms of visual category perception in human brain.

## Results

### Feature importance allows to separate out the neural signals that are predictive of perceptual categorization from the mixture of stimulus-induced responses

To identify spectrotemporal features that are characteristic of automatic perceptual categorization of a particular category we relied on time-frequency (TF) maps of the neural responses of intracranially implanted electrodes. Out of the total set of 11321 probes 11094 (98%) were responsive (see the Methods section on processing of neural data for details) to the stimuli from at least one of the categories. On one hand this provides us with abundance of data, on the other raises the question whether all of that activity was relevant to the processes that encode and process visual input.

**Figure 1:**
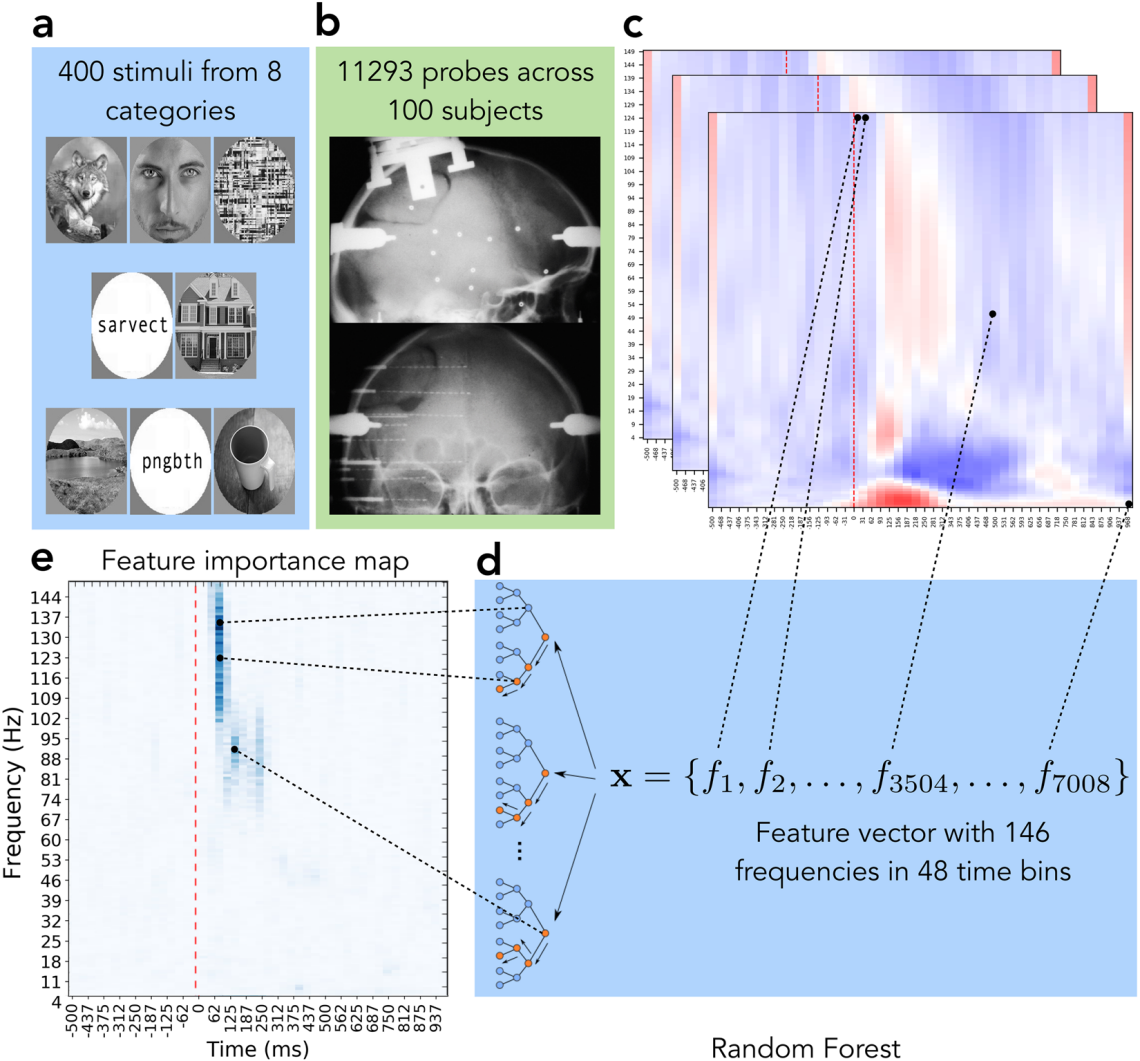
Major steps of the data processing pipeline. (**a**) Image stimuli from 8 categories were presented to test subjects. (**b**) Human brain responses to images were recorded with deep intracranial electrodes. (**c**) LFP signals were preprocessed and transformed into time-frequency domain. (**d**) Random Forest models were trained to decode image category from each electrode’s activity. (**e**) Feature importances of each model were calculated to identify the region on each electrode’s activity map that was relevant to visual object recognition. Notice how the final results on panel (e) tell us that high gamma activity in 90–120 ms window and the subsequent activity in the low gamma range in 120–250 ms window are the only bands and time windows in that particular electrode’s activity that are relevant for the classification task, while the spectrogram on panel **c** also shows that there was activity in early theta, beta and low gamma bands. Our analysis revealed that not all activity was relevant (or useful) for the classification of an object and showed which parts of the activity are actually playing the role in the process.

Training a decoding model (see the Methods section on Random Forest as decoding model and the illustration on Fig. 1) for each of the probes allowed us to dissociate the *predictive probes* that exhibited activity that was useful for decoding from the rest of the *responsive probes* that did not carry such activity.

**Figure 2:**
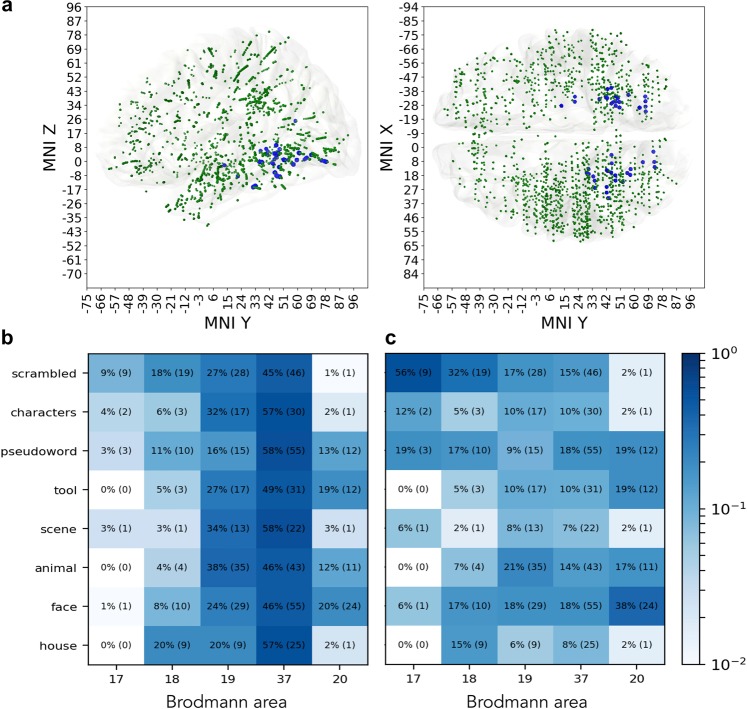
Distribution of predictive probes. (**a**) Green markers indicate all of the probes that were responsive to stimuli from the house category. Blue markers indicate only the predictive probes that carry information that is relevant to decoding the neural response as reaction to house stimulus. The house category was selected for this figure as an illustrative example, similar effect can be seen across all 8 categories, please see the Supplementary Materials repository for the visualization of the distributions of responsive and predictive probes in all eight categories. (**b**) Distribution of predictive probes over areas within each category (each row sums up to 100%). Color shows the percentage across categories. (**c**) Distribution of predictive probes over a category within each area (each column sums up to 100%). Color shows the percentage across areas.

Green markers on Fig. 2a show the set of probes that are responsive to the house category, while the blue markers are the probes that are predictive of that category (4.8%, 535 probes). Figure showing the distribution of responsive and predictive probes for other categories are available in Supplementary Materials repository. Decoding models built on the neural responses of the predictive probes were successful at classifying at least one perceptual category (${{\rm{F}}}_{1} > 0.39$ for one or more classes), focusing on them in our further analysis allowed to work only with the locations that carry information relevant to the task of perceptual categorization.

Predictive probes had a heterogeneous distribution in the brain, yet remained mostly concentrated in visual cortices and inferior temporal regions (76%), from BA17 to BA20, including early visual areas (BA 18, 19), fusiform gyrus (BA 37) and inferior temporal cortex (BA 20). A majority of the predictive probes were in fusiform cortex (average of 52% over all categories, Fig. 2b), followed by BA 19 (27%), across all category networks. The detailed numbers of the distribution of predictive and responsive probes across categories and Brodmann areas can be found in Table 1 of the Supplementary Materials Section.

Within the primary visual cortex, BA 17 and 18, the scrambled was the stimulus that elicited most predictive probes (28) amongst all stimulus categories (Fig. 2c), followed by pseudowords (13). Probes predictive of faces were mostly concentrated in BA19, BA37 and BA20 (72%, 108 out of 150). The low number of predictive probes in area 17 is explained by the fact that less than 1% of the implantation sites in the original dataset were located in primary visual cortex.

Previous studies have shown that perceptual category-selective networks are located in occipito-temporal cortex^4,6,12^. To test whether predictive power of the Random Forest model trained to decode activity of probes is coherent with known functional processing by cortical networks we evaluated the selectivity of the predictive power in three known functional networks: Fusiform Face Area (FFA)^1^, Visual Word Form Area (VWFA)^7^ and Parahippocampal Place Area (PPA)^39^. We checked whether the probes located in each of these areas and the Random Forest model trained on these probe’s activity to discriminate between 8 categories produces the highest predictive power for the category for which this area is known to be selective. Probes in FFA are associated with facial recognition and encoding facial information^40–44^ and thus we expect their activity to be predictive of the face category, probes in VWFA should be predictive of characters and pseudowords categories^42,45,46^ and probes in PPA should be responsive to scenes and houses^39,47–49^.

There were 12 probes in the FFA that were significantly (permutation test $p < 1e-4$) predictive (classification score ${{\rm{F}}}_{1} > 0.39$) of a category: 5 were predictive of faces, 4 of animals (which mostly have faces on the image), 2 of pseudowords and 1 of scrambled images. Most probes that were in FFA and were predictive, carried information of the categories containing facial features.

There were 8 probes in the VWFA that were predictive of a category: 5 were predictive of pseudowords, 2 of characters and 1 of faces. This points to the fact that the predictive probes in VWFA are predictive of the stimuli with written characters on them. These results confirm that predictive power of a Random Forest model trained on probes activity in VWFA reflects the functional role known to be carried by this area.

For probes in the PPA results were less selective. There were 23 probes inside that area that were predictive of a category: 5 were predictive of houses, 4 of scenes, 5 of characters, 5 of scrambled images, 2 of tools and 2 of pseudowords. The probes from PPA predicted not only houses and scenes, but also other categories. However, houses and scenes were among the categories that the probes from PPA were able to identify successfully in highest proportion as compared to the other categories.

These confirmatory findings give credibility to the methodology by which the probes that are identified as predictive of a certain category are involved in the processing of the stimuli that belong to that category.

Training per-probe decoding models not only allowed us to identify the predictive locations, but also to apply feature importance analysis to decoding models trained on local activity. Computing the feature importance across the time-frequency map (4–150 Hz and −500 to 1000 ms) allowed us to see which parts of neural activity are crucial for the decoding. Overlaying the importance over time-frequency map showed at which frequencies and at what times the activity that was important for the algorithm has occurred. This can be applied both on aggregated level, where the importance map is averaged over probes, and on individual probe level. “Feature importance of the analysis of task-relevant neural activity” part of the Methods section explains the application of probe importance map to filter irrelevant activity and obtain spectrotemporal signature of a particular category on a particular probe. Now we can use the feature importance map as a mask and perform the analysis of the activity itself, focusing only on the relevant parts of it. When applicable, this methodology helps to filter out irrelevant activity and allows to focus on the activity that is important to the scientific question under investigation.

**Figure 3:**
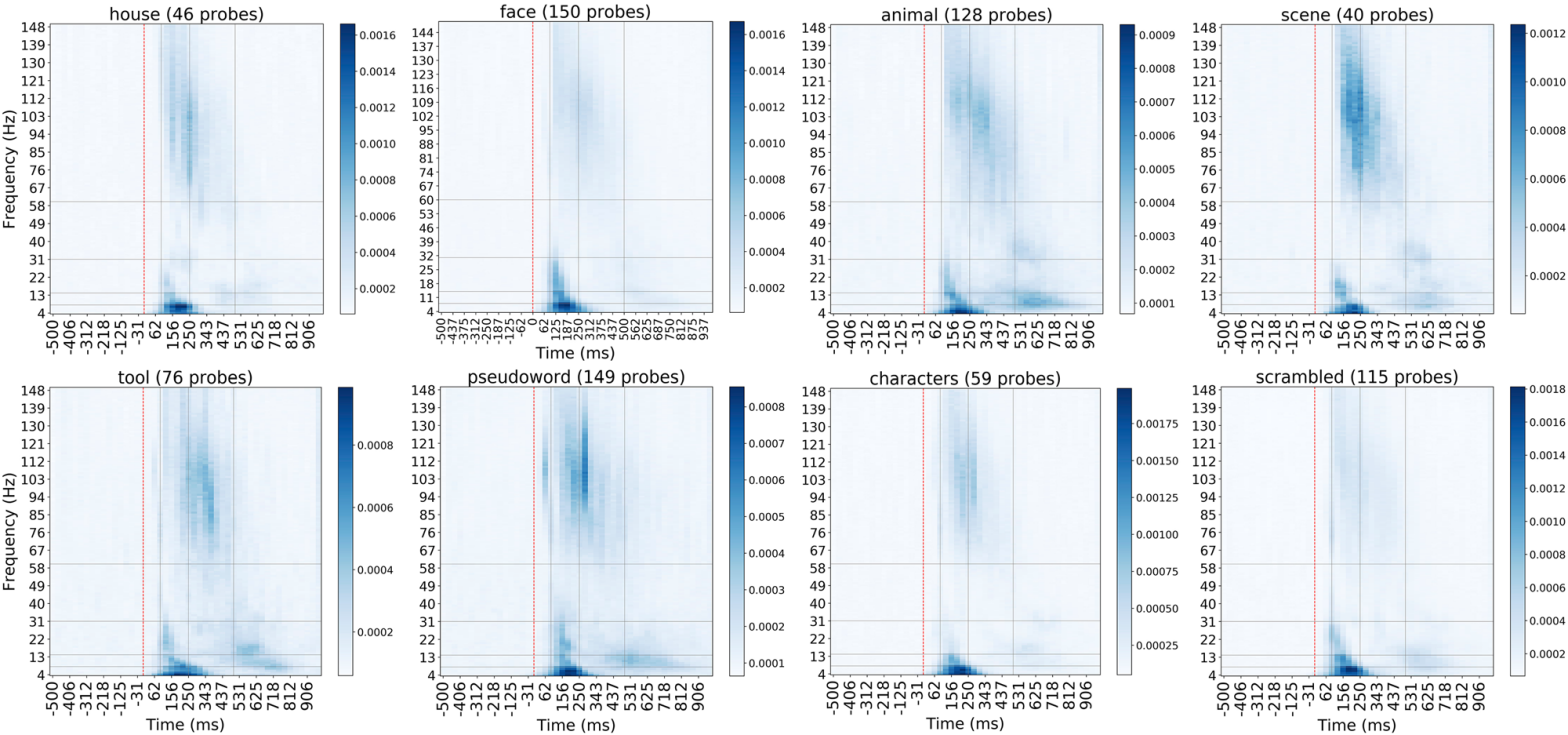
Average importance map of each of eight categories over probes predictive of that category. The color shows the relative importance of each spectrotemporal feature, indicating how informative that particular feature was for the task of decoding.

We took an average over importance maps of all individual probes within each category to obtain the global picture of where the category-specific activity lies in time and frequency space. Figure 3 visualizes average importance maps of each of eight categories and allows to single out the spectrotemporal signatures that are unique to specific categories and those that are less selective. From these importance maps we notice that certain TF components are distinctly present per category, as for example high (significantly higher than 81 out of 112 regions of interest, Mann-Whitney U $p < 8.9{\rm{e}}-7$, corrected) importance of the transient theta activity (theta burst) in all categories, or the almost absence of importance of broadband gamma (significantly lower than 10 out of 12 other regions of interest, Mann-Whitney U $p < 8.3{\rm{e}}-5$, corrected) in the control scrambled condition.

In the following sections we expand our analysis to the comparison of the feature maps and analyzing the activity under the regions that the method identifies as important.

### Polypredictive and monopredictive probes

**Figure 4:**
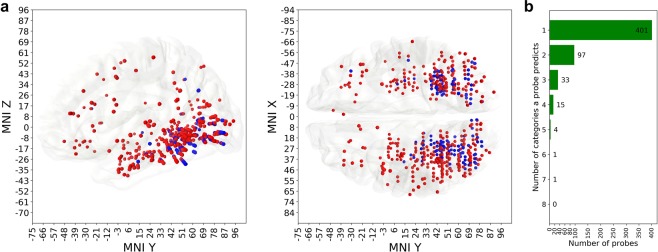
Anatomical distribution of mono- and polypredictive locations. (**a**) Red markers are the locations of monopredictive probes, blue markers are the locations of polypredictive ones. Polypredictive probes (145 unique locations) are mostly confined to visual areas and temporal lobe (both parts of the ventral stream), while monopredictive (specialized, 401 unique locations) probes are, in addition to visual areas, also found in frontal and parietal cortical structures. (**b**) The histogram shows how many categories are predictable by how many probes.

The analysis revealed two types of neural locations: *polypredictive* probes are predictive of multiple visual categories, while *monopredictive* are useful for decoding only one out of 8 different types of stimuli revealing a high degree of specialization (Fig. 4b). We considered a probe to be predictive of a category if cross-validation F_1_ score for that category was higher than 0.39 (see the Methods for details on the threshold selection), which is a stricter condition than above-chance criterion (${{\rm{F}}}_{1} > 0.125$). We use the term ‘predictive‘ to highlight the difference in meaning with the classical terms ‘selective‘ and ‘responsive‘. Both these terms describe the post-stimulus activity of an electrode in terms of the relationship of the post-stimulus response to the pre-stimulus baseline. The relevance of the electrode’s activity to the classification task, however, is measured in this work using classifier’s prediction score.

Figure 4a shows that polypredictive probes reside mainly (94%, 136 out of 145) in posterior occipital and posterior temporal, while the monopredictive probes extend, in addition to occupying similar posterior occipital and temporal locations, to frontal cortex (92%, 45 out of 49 probes in this area are monopredictive) and anterior temporal cortex (88%, 51 out of 58 probes). Both mono- and polypredictive probes are also observed in parietal cortex. Monopredictive probes that extend beyond ventral stream and temporal cortex pertain to the following perceptual categories: faces (orbitofrontal cortex), animals and pseudowords (dorsofrontal cortex, inferior frontolateral cortex, premotor cortex), and, to a smaller extent, scrambled images (prefrontal cortex).

**Figure 5:**
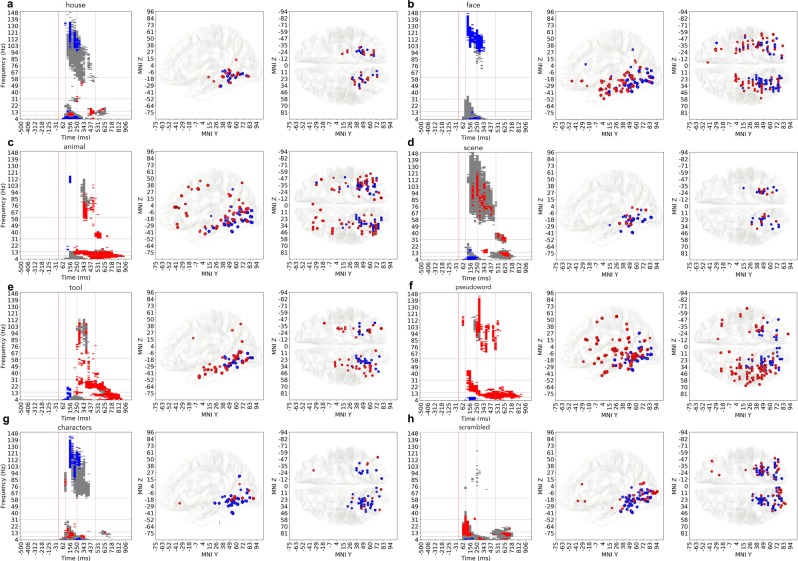
Statistically significant differences between the importance of monopredictive and polypredictive probes’ activity. Gray regions indicate the areas of TF spectrum where both monopredictive and polypredictive probes exhibit important (2*σ* from the mean) activity. On top of it, if one of the groups is statistically significantly (4*σ* difference) more important than another, the region is colored with blue (polypredictive) or red (monopredictive) to show which of two kinds of neural specialization dominates this TF region in terms of importance. For example decoding of scenes (d) involves early theta activity of polypredictive (blue) probes, followed broadband gamma activity that is significantly important (gray), and is slightly dominated by monopredictive (red) probes, then followed by late alpha activity produced predominantly by monopredictive neural locations. (**a**) house, (**b**) face, (**c**) animal, (**d**) scene, (**e**) tool, (**f**) pseudoword, (**g**) characters, (**f**) scrambled.

The unique association of specific TF feature importance components with either polypredictive and monopredictive probes was category specific, as shown in Fig. 5a–h. While all of the data presented on these figures shows statistically significant differences between monopredictive and polypredictive neural locations, we will focus only on a few that were supported by the strongest signal in the data. For face stimuli, most of the feature importance in the early broadband gamma response was significantly (4*σ*) higher in polypredictive probes as compared to monopredictive probes, indicating that the most useful information for distinguishing faces from other visual categories is coded in that region of time-frequency space and is carried by polypredictive probes (Fig. 5b). Decoding of animals and tools relied on the activity patterns produced by monopredictive neural locations in late broadband gamma range (>300 ms) and in even later (350–600 ms) alpha/beta range, with very little involvement of the activity of polypredictive probes. Scenes and houses also show strong feature importance in late alpha and beta band responses of monopredictive probes ($4\sigma$ higher). Interestingly, for characters (Fig. 5g), feature importance in the early broadband gamma range was dominant for polypredictive probes (4*σ* higher than monopredictive), while the opposite was true for the pseudowords (Fig. 5f) – the late broadband gamma revealed to be dominant for monopredictive probes, also note the difference in the anatomical locations that were the most useful for the decoding of the pseudowords compared to the locations that were useful for decoding characters. Pseudowords also elicited a significantly stronger TF feature importance in monopredictive probes in late (350–750 ms) low-frequency (4–12 Hz) range, similar to animal and tool stimulus categories. Finally, an interesting observation was that animals and faces share most of their polypredictive probes (51%) indicating a large overlap of categorization networks of these two categories.

### Further decomposition of important activity reveals clusters of distinct time-frequency patterns

**Figure 6:**
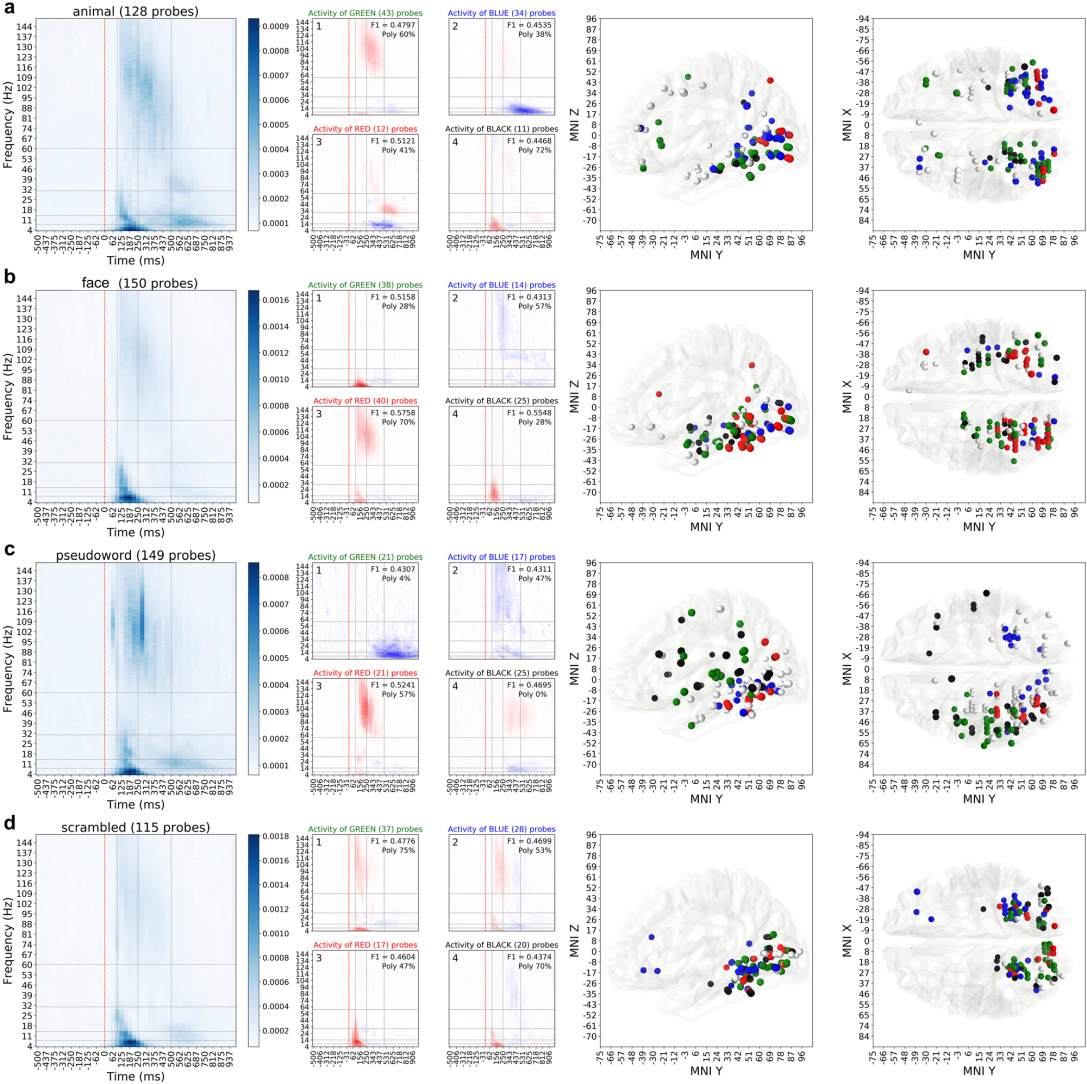
Detailed analysis of spectral activity of (**a**) animals, (**b**) faces, (**c**) pseudowords and (**d**) scrambled images. For this figure we have selected the four categories that contained the most interesting observations, same visualization of activity importance, cluster assignations and the anatomical locations of those clusters for other four categories are available in the Supplementary Materials repository. Leftmost column contains the importance maps extracted from Random Forest models and shows where in time and frequency the important activity is. Second column visualizes the four largest (by the number of recording sites) clusters of activity patterns inside those spectrotemporal regions that are deemed important. The numbers in the top right corner of each cluster’s activity pattern show the average predictive power (F_1_ score) of the probes in that cluster and proportion of polypredictive locations that exhibited this particular pattern of activity. Note how every cluster has a designated color: green, blue, red or black. This color of the cluster matches the color of MNI location markers in the last two columns, that show sagittal and dorsal views of the brain. White markers show the probes that have predictive power, but their activity pattern does not belong to any of the four major clusters.

We ran clustering analysis of the probes predictive of a category based on their activity to see which probes in the category-network behave in a similar way. Left column of Fig. 6 shows an averaged feature importance map for a given category. We look into the regions of the time-frequency map that are indicated as important by the feature importance map, extract baseline-normalized activity in those regions and cluster the probes according to that activity using hierarchical complete linkage clustering with cosine distance (see the Methods section on hierarchical clustering for details). The second column of Fig. 6 shows the activity of four most populated clusters for each category. Each cluster represents the activity pattern exhibited by the probes in that cluster. Only the probes whose activity had predictive power (${{\rm{F}}}_{1} > 0.39$) are included in this analysis. As the final step we identified the anatomical locations of the probes from each cluster to see whether difference in the activity patterns could be attributed to the functional regions of the brain. The visualization of this step in the last two columns of Fig. 6.

This analysis allowed us make a number of *global* and *category-specific* observations. The set of visual categories presented in our data is diverse enough to consider category-specific findings to be general and emerge under any comparable set of visual stimuli.

The first global observation was that it is not only broadband gamma activity that is useful for the decoder’s (Random Forest) performance, but low-frequency activity also contributed significantly (41% of predictive probes exhibited only low-frequency activity in the regions of importance), sometimes overshadowing the activity of higher frequency bands altogether (for face and scrambled stimuli low frequency activity was significantly more important than broadband gamma activity, Mann-Whitney U test $p < 1{\rm{e}}-7$, corrected). Most clusters were composed of a combination of low and high-frequency components (Fig. 6, second column) and were mostly (87%) located in occipito-temporal cortices, though some electrodes in parietal and frontal cortex (7%) also appeared to contribute with predictive responses in the decoding process (Fig. 6, two right columns), especially for such stimulus categories as animal and pseudoword.

The second observation spanning across all categories was that the classifier used not only the increases in power to perform the classification, but also relied on power decreases in different brain networks (7 out of 32 dominant activity clusters consisted solely from the activity patterns characterized by power decrease). The most prominent examples are the clusters faces-2 (Fig. 6b), animals-2 (Fig. 6a), tools-2, pseudowords-1, pseudowords-2 (Fig. 6c), scrambled-1 and scrambled-2 (Fig. 6d). For example, to decode face or pseudowords from the activity of the blue cluster network, the RF classifier used broadband gamma power decreases located in posterior inferior temporal cortex and inferior occipital gyrus. None of the probes for which the decrease in activity was identified as important for decoding were located in classically defined Default Mode Network^50,51^.

Across all categories, the earliest component that often appeared in clusters was the brief power increase (mean non-zero power increase was 2.8 times the baseline in the region of interest) in the low-frequency interval (4–25 Hz), which for one group of probes can be associated to an almost instantaneous broadband gamma power increase (6b, cluster 3, mean broadband gamma increase of 1.9 times the baseline), but remains the only source of important activity for another group of probes (6b, cluster 1).

Studying the anatomical locations of the probes belonging to different clusters of activity revealed interesting observations. Figure 6c, pseudowords, clusters 1 and 3 show a clear example how clustering by activity patterns leads to assigning the probes into functionally different anatomical areas. The gamma-band increase signature captured by cluster 3 occurs only in the left hemisphere (red markers on Fig. 6c), the late theta-alpha power decrease captured by cluster 1 also occurs only in the left hemisphere (green markers) and is spatially clearly distinct from probes in cluster 3. Because it is known that pseudoword stimuli elicit top-down language-related (orthographic, phonological and semantic) analysis, which elicits highly left-lateralized networks identifiable in iEEG recordings^52,53^, we know that this observation reflects a functional brain process. This dissociation in both the spectrotemporal and anatomical domains provides us with valuable data on the locations and associated activity patterns emerging during automatic perceptual categorization and highlights the benefit of disentangling the activity into functionally and anatomically disassociated clusters.

**Figure 7:**
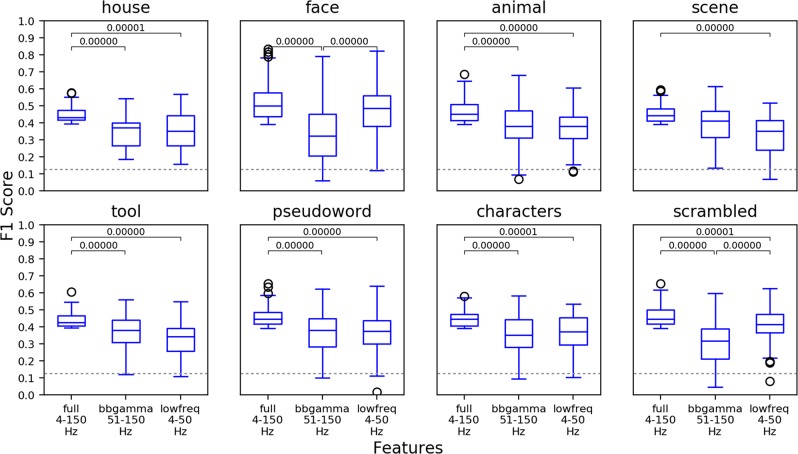
Comparison of predictive power of the electrodes from three different sets of features: full spectrum (4–150 Hz), broadband gamma alone (50–150 Hz) and lower frequencies alone (4–50 Hz) across categories. The bracket with the p-value indicates a significant difference according to Mann–Whitney U test. The dashed line indicates change classification level for an 8-class problem.

Finally, the relevance of the different components in the TF domain for the Random Forest classification process was assessed. Specifically, we tested whether the activity in the broadband gamma range, commonly present on most clusters across categories, is in general the most valuable neural signature for category networks as compared to the low-frequency parts of the spectrum. To test whether broadband gamma was solely the most informative frequency interval we statistically compared predictive power of three intervals: broadband gamma (50–150 Hz), low-frequency (4–50 Hz) and full spectrum (4–150 Hz). Overall, across 7 perceptual categories out of 8 (except for scenes), using the full spectrum was more informative than using the broadband gamma interval or the low-frequency interval alone (Mann–Whitney U test, $p < 0.001563$, corrected to the number of clusters compared, see Fig. 7), which is in line with the results reported by^54^. Importantly, for scrambled images and faces the broadband gamma carried *less* (Mann-Whitney U $p < 1{\rm{e}}-7$, corrected) decoding-relevant information than the lower frequencies.

## Discussion

In the present work we explored the bottom-up approach to the analysis of human intracerebral neural activity. Due to a rich dataset and powerful methodology we were able to uncover facts about neural processing of automatic visual categorization that we would not necessarily address in a hypothesis-driven study. We trained a machine learning model to decode 8 different perceptual categories from human intracerebral neural spectral activity and analyzed the resulting statistical model to expose what information does the model rely on in order to make decoding decisions. We then drew the parallels between the patterns of neural activity that the machine learning algorithm deemed important and the functional role of that activity for the task of decoding perceptual categories in human brain. This allowed us to distinguish between the spectral activity that is relevant for the task from the activity that is not. The study was conducted using a large dataset consisting of 4528400 local field potential recordings, from a cohort of 100 human patients whose combined electrode implantations spanned across all major brain cortical areas. The decoding process confronted neural responses elicited by 8 different perceptual categories (from 8 different stimulus sets), which allowed obtaining a high resolution in the dissociation between neural response patterns across categories. All classifications were operated on a broad frequency spectrum ranging from 4 to 150 Hz and allowed to distinguish degrees of selectivity of neural responses and which spectral components most strongly enable this selectivity. Previous works have shown where and when perceptual category information can be decoded from the human brain, our study adds to that line of research by identifying spectrotemporal patterns that contribute to category decoding without the need to formulate a priori hypothesis on which spectral components and at which times are worth investigating.

The classifier model first allowed us to globally identify two types of neural responses: those that were predictive of a certain category and those that did not predict any category despite eliciting strong amplitude modulation across multiple frequency bands. Surprisingly, when comparing the level of predictability of probe responses we found that only 4.8% of the responsive probes were predictive of a category. This very low percentage highlights an important fact regarding the level of “selectivity” of a neural responses. In this decoding approach, the level of single-probe neural response selectivity depends on the diversity and overall quantity of the comparison/reference group to which it is compared to. Stimulus-induced neural signal selectivity is thus a graded quality that can be assessed through multiple comparisons with a broad variety of stimulation conditions. This result also implies that although any stimulus can elicit a local neural response throughout the cerebral cortex, in the light of our results, there is a high probability of it being non-predictive of any of the categories or being polypredictive of several categories at once.

In line with a vast literature on the localization of category related networks^1–7,12–14^ predictive probes concentrated mostly in the inferior temporal cortex, namely the fusiform gyrus (BA 37), yet surprisingly for some categories, probes in primary visual cortex were also predictive of these categories. This effect is probably related to the specifics of the physical content of certain images that uniquely characterize certain categories amongst all others, as for example the content in high-contrast edge information in scrambled and written text stimuli. The existence of polypredictive neural locations could be an important finding in the context of the debate concerning the functional organization of the inferior temporal cortex. Specifically, this finding contradicts a modular organisation in which each region is involved in processing a single category^55^, and instead favours a more distributed, overlapping representation of categories across the IT^56^.

Predictive probes were subsequently classified according to their level of selectivity towards a single or multiple visual categories. Polypredictive probes (36%) clustered in visual cortices and inferior temporal cortex and were associated with early spectral components (<300 ms) such as broadband gamma power increases and a transient theta burst shortly after stimulus presentation. Monopredictive probes (64%) were abundant in these same regions, but extending uniquely in frontal, parietal, superior temporal and anterior limbic cortex. Their activity was strongly associated with the later (>300 ms) time and with power suppression of spectral importance features, versus baseline, in the theta (4–7 Hz), alpha (8–15 Hz) and beta bands (16–40 Hz). In a subgroup of probes the associated power suppression of the feature importances extended into the broad gamma band (50–150 Hz).

Importantly, the capacity to ascribe category selectivity to predictive probes (mono vs polypredictive probes) arises from the fact that the decoding model was trained to discriminate between all 8 categories simultaneously. The separation between mono and polypredictive probes revealed specific effects in terms of network localization and time-frequency components. The high concentration of polypredictive probes (and local networks) in early visual cortices, from primary visual cortex up to inferior temporal cortex is coherent with the idea that networks in the ventral visual stream progressively integrate more complex features into object representations, thus becoming progressively more selective, and converge within median temporal lobe to more stimulus-invariant representations^15^. This progressive information integration by spectral features of neuronal responses across the visual hierarchy has been recently connected with the computations carried out by deep convolutional neural networks trained to solve the task of visual recognition^57^.

Globally, the random forest data classification provided results that are coherent with current knowledge on 1) the implication of networks located in visual cortex and inferior temporal cortex in processing visual categories, 2) the timing of object categorization in the human brain and 3) the role of broadband gamma responses in processing category-selective information within these networks. Previous studies have shown that certain stimulus categories elicit clustered cortical responses of highly localized networks in the occipito-temporal ventral stream such as the fusiform-face-area (FFA) and the visual-word-form area (VWFA)^1,7^. Yet, other studies have broadened this scope by showing that certain categories, as for example faces, rely on the involvement of a larger brain-wide distributed network^34,58^. Our classification analysis shows that the spatial extent of this network distribution is category specific, certain stimuli eliciting larger network responses, such as for faces, animals and pseudowords, as compared to scenes, houses and scrambled images which concentrate in the fusiform cortex, the parahippocampal cortex and primary visual cortex respectively.

Our results largely agree with previous works trying to decode visual object categories over time with MEG^59,60^ or intracranial recordings^61^. All these studies converge on the result that perceptual categories can be decoded from human brain signals as early as 100 ms. Our current work goes a step beyond these previous investigations by demonstrating which spectral components underlie this fast decoding. Previous intracranial studies have also shown that broadband gamma is modulated by information about object categories^34,37,38^. Moreover, broadband gamma has been suggested as a proxy to population spiking output activity^30,62–64^. It has since then been considered as a hallmark of local population processing^31^. Our classification results however show that broadband gamma is not the sole selectivity marker of functional neural processing, and that higher decoding accuracy can be achieved by including low-frequency components of the spectrum. For certain stimulus categories, as scrambled images, the broadband gamma range is even outperformed by the predictive power of the low-frequency range.

To understand which spectral components play a specific role in stimulus categorization we analyzed the decision process that drives the decoding model and identified the combined spectrotemporal regions that are informative for the output of the random forest classification procedure. This allowed us 1) to identify the category-selective spectral components of high importance for the automatic visual categorization process, and 2) identify the correlates functional involvement of positive as well as negative power modulations (increases and decreases versus baseline) in early and late time windows of neural processing involved in visual categorization.

While the distinctive activity of polypredictive neural locations is mostly reflected by early TF components (i.e. broadband gamma and theta burst in faces), the sustained decrease in power in the alpha/beta band was extended in space and time. This process is probably dependent on the degree of difficulty for the networks in reaching a perceptual decision and which appeals to the involvement of top-down processing required to resolve perceptual ambiguity elicited by the different stimulus categories. For example, animal and tool stimuli are highly diverse in their physical image structure, as compared to face stimuli. This affects the efficiency of bottom-up process in extracting category information, often associated with increase in gamma activity, and probably in parallel triggers top-down processes through selective activity modulation in low-frequency channels^65^. In our data, this latter phenomenon could be mirrored by a decrease of predictive power in the low-frequency range. Studies have shown that power modulations reflect changes in network connectivity^66^ and that top-down processes, eliciting a decrease in power in the alpha-beta band, are accompanied by an increase in distant network connectivity^67^.

Finally, we also show that certain probes elicit decreased broadband gamma responses (versus baseline) while representing a significant feature importance for the classification model. It has been shown that neural activity in the Default Mode Network can be negatively modulated by attending sensory stimulation^50^, and intracranial studies have found that this was reflected by decreases (versus baseline) in the broad gamma range^68–70^. Here we found no evidence of such power decreases in probes located in the DMN^50^. However, the random forest classifier singled-out broad spectral patterns of power decreases at probes located in visual regions and beyond for categories faces, pseudowords and characters. This is the first time, to our knowledge, that power decreases in the broadband gamma range outside the DMN have been associated with highly functional neural signal classification of perceptual categories. Their functional significance should be studied in the future as they could reflect an important phenomenon of communication regulation between networks during perceptual decision making of visual categories.

In this work we studied the information that allows local cortical populations of neurons to make predictions of the category of a visual stimulus. Expanding on this work by including more subject data in the future might allow us to make a transition from the observations of local activity and the analysis of its role to being able to detect signatures of global decision-making processes. It is possible that these signatures would be reflected in specific spectral fingerprints as many classic theories would suggest^22,71–73^. The methodology proposed in this study can facilitate the search of those fingerprints without the need to formulate a priori hypothesis about which spectrotemporal components are worth investigating.

## Methods

### Patients and recordings

100 patients of either gender (49 females, 51 males) of the mean age of 33.18 ± 10.13 years with drug-resistant partial epilepsy and candidates for surgery were considered in this study and recruited from Neurological Hospitals in Grenoble and Lyon (France). All patients were stereotactically implanted with multi-lead EEG depth electrodes (DIXI Medical, Besançon, France). All participants provided written informed consent, and the experimental procedures were approved by local ethical committee of Grenoble hospital (CPP Sud-Est V 09-CHU-12). All methods were performed in accordance with the guidelines and regulations of the ethical committee of Grenoble hospital. Recording sites were selected solely according to clinical indications, with no reference to the current experiment. All patients had normal or corrected to normal vision.

#### Electrode implantation

11 to 15 semi-rigid electrodes were implanted per patient. Each electrode had a diameter of 0.8 mm and was comprised of 10 or 15 contacts of 2 mm length, depending on the target region, 1.5 mm apart. The coordinates of each electrode contact with their stereotactic scheme were used to anatomically localize the contacts using the proportional atlas of Talairach and Tournoux^74^, after a linear scale adjustment to correct size differences between the patient’s brain and the Talairach model. These locations were further confirmed by overlaying a post-implantation MRI scan (showing contact sites) with a pre-implantation structural MRI with VOXIM^®^ (IVS Solutions, Chemnitz, Germany), allowing direct visualization of contact sites relative to brain anatomy.

All patients voluntarily participated in a series of short experiments to identify local functional responses at the recorded sites^34^. The results presented here were obtained from a test exploring visual recognition. All data were recorded using approximately 120 implanted depth electrode contacts per patient using SD LTM Express, Micromed system for signal acquisition with a sampling rate of 512 Hz, high-pass filter 0.15 Hz, low-pass filter 500 Hz. Data were obtained from a total of 11321 recording sites.

#### Stimuli and task

The visual recognition task lasted for about 15 minutes. Patients were instructed to press a button each time a picture of a fruit appeared on screen (visual oddball paradigm). Only non-target stimuli were used in this work, the target fruit category was excluded from the analysis. Non-target stimuli consisted of pictures of objects of eight possible categories: houses, faces, animals, scenes, tools, pseudo words, consonant strings, and scrambled images. Natural images for the appropriate categories were obtained from the public domain on the Internet. Pseudoword and character images were generated by inserting a desired string onto a white background. All the included stimuli had the same average luminance. All categories were presented within an oval aperture of 2 × 3 in visual angle (illustrated on Fig. 1a) at a distance of 70–90 cm using NeuroBehavioral Systems (NBS) Presentation^®^ software. Stimuli were presented for a duration of 200 ms every 1000–1200 ms in series of 5 pictures interleaved by 3 second pause periods during which patients could freely blink. Patients reported the detection of a target through a right-hand button press and were given feedback of their performance after each report. A 2 second delay was placed after each button press before presenting the follow-up stimulus in order to avoid mixing signals related to motor action with signals from stimulus presentation. Altogether, responses to 400 unique natural images were measured per subject, 50 from each category.

### Processing of neural data

The analyzed dataset consisted of $4528400$ local field potential (LFP) recordings – responses from 11321 recording sites to 400 stimuli. To remove the artifacts the signals were linearly detrended and the recordings that contained values ≥ 10*σ*_*images*_, where *σ*_*images*_ is the standard deviation of voltage values (in the time window from −500 ms to 1000 ms) of that particular probe over all stimuli, were excluded from data. All electrodes were re-referenced to a bipolar reference and the reference electrodes were excluded from the analysis. The signal was segmented in the range from −500 ms to 1000 ms, where 0 marks the moment when the stimulus was shown. The −500 to −100 ms time window served as a baseline.

To quantify the power modulation of the signals across time and frequency we used standard time-frequency (TF) wavelet decomposition^75^. The signal $s(t)$ was convoluted with a complex Morlet wavelet $w(t,{f}_{0})$, which has Gaussian shape in time $({\sigma }_{t})$ and frequency $({\sigma }_{f})$ around a central frequency ${f}_{0}$ and defined by ${\sigma }_{f}=1/2\pi {\sigma }_{t}$ and a normalization factor. To achieve good time and frequency resolution over all frequencies we slowly increased the number of wavelet cycles with frequency, $\frac{{f}_{0}}{{\sigma }_{f}}$ was set to: 6 for high (61–150 Hz) and low (31–60 Hz) gamma, 5 for beta (15–30 Hz), 4 for alpha (9–14 Hz) and 3 for theta (4–8 Hz) frequency ranges. This method allowed to obtain better frequency resolution than applying a constant cycle length^76^. The square norm of the convolution results in a time-varying representation of spectral power, given by: $P(t,{f}_{0})=|w(t,{f}_{0})\cdot s(t){|}^{2}$. Baseline normalization was performed by dividing the average power after stimulus onset ($0$ to $1000$ ms) in each frequency by the average power of that frequency in the baseline window (−500 to −100 ms). Each LFP recording was transformed from 768 data points (1.5 seconds of voltage readings at 512 Hz sampling rate) into a matrix of size 146 × 48 where each row represents a $1$ Hz frequency band from 4 Hz to 150 Hz and columns represent 31.25 ms time bins. Value in each cell of that matrix is the power of that specific frequency averaged over 16 time points.

Further analysis was done only on the electrodes that were responsive to the visual task. In each frequency band we compared each electrode’s average post-stimulus band power to the average baseline power with a Wilcoxon signed-rank test for matched-pairs. Only the probes that showed a post-stimulus response that is statistically significantly (p-value ≤ 0.005, corrected for multiple comparisons with the false discovery rate (FDR) procedure^77^) different from the baseline response in at least two frequencies were preserved for future analysis. Please note that eliciting a significant response in at least 2 out of 146 frequencies is a relaxed requirement. The use of such a relaxed criterion allowed us to include into analysis not only the areas that had a strong response in the visual areas, but also the responses from other brain areas that might reflect downstream processes related to automatic perceptual categorization. This was possible due to the fact that the proposed method, given sufficiently large dataset, will not be hindered by the additional volume of irrelevant data and is able to detect narrow phenomena even in the large corpus of data.

To anatomically localize the source of each signal in subject’s brain each electrode’s MNI coordinates were mapped to a corresponding Brodmann brain area^78^ using Brodmann area atlas from MRICron^79^ software.

To confirm that probe’s predictiveness of a certain category implies that the probe belongs to the network selective of that category we ran a set of experiments on three well-known functional areas: Fusiform Face Area (FFA)^1^, Visual Word Form Area (VWFA)^7^ and Parahippocampal Place Area (PPA). Following Montreal Neurological Institute (MNI) coordinates of FFA reported in^80^ and^81^ we defined FFA bounding box as $x\in [\,-\,44,-\,38]$, $y\in [\,-\,61,-\,50]$, $z\in [\,-\,24,-\,15]$ in the left hemisphere and $x\in [36,43]$, $y\in [\,-\,55,-\,49]$, $z\in [\,-\,25,-\,13]$ in the right hemisphere. Based on the Table 1 from^82^ we defined VWFA area as MNI bounding box $x\in [\,-\,50,-\,38]$, $y\in [\,-\,61,-\,50]$, $z\in [\,-\,30,-\,16]$ in the left hemisphere. From MNI coordinates reported in^83^ and^84,85^ we defined PPA bounding box to be $x\in [\,-\,31,-\,22]$, $y\in [\,-\,55,-\,49]$, $z\in [\,-\,12,-\,6]$ in the left hemisphere and $x\in [24,32]$, $y\in [\,-\,54,-\,45]$, $z\in [\,-\,12,-\,6]$ in the right hemisphere.

### Random Forest as a decoding model

A Random Forest^86^ is a collection of decision trees, where each tree gets to operate on a subset of features. Each tree is assigned a random set of features and it has to find the decision boundaries on those features that lead to best classification performance. At each branching point the algorithm must decide using which feature will be most efficient in terms of reducing the entropy of class assignations to the data points under current branch of the decision tree. To achieve that, the feature that is most useful will be selected first and will be responsible for largest information gain. For example, if the activity of a probe at 52 Hz at 340 ms is high when a subject is presented with a face and low for all other categories, decision tree will use that fact and rely on the “52 Hz at 340 ms” feature, thus assigning it some importance. How high the importance of a feature is will depend on how well does this feature distinguish faces from all other categories. As Random Forest is a collection of trees and the same feature will end up being included into several different trees, being important in many trees contributes to the overall importance of a feature (for the exact computation see the section on feature importance below).

We treated each electrode’s responses as a separate dataset consisting of 400 data points (one per stimulus image), and 7008 features – time-frequency transformation of LFP response into 146 frequencies and 48 time bins. For each electrode we trained a Random Forest with 3000 trees and used 5-fold cross-validation to measure the predictive power of the neural activity recorded by each of the electrodes. Per-class F_1_ score, a harmonic mean of precision and recall of a statistical model, provides us with a metric of success of the classification. The parameters were selected by performing informal parameter search. Random Forest was the algorithm of choice in our analysis due to its previous application to spectrotemporal features^87^ and, more importantly, due to the interpretability of the resulting models. Highly interpretable structure of the resulting decision trees allowed us to follow the decision-making process that led each particular model to a decoding decision, and to estimate each feature’s contribution to this process, allowing to identify and characterize category-specific spectral signatures. We used scikit-learn^88^ implementation of the above-mentioned methods with default parameters unless indicated otherwise.

As the first step of the decoding analysis we estimated which of 11321 electrodes have predictive power. For that we split each electrode’s 400-sample dataset into 320 samples for training and 80 for prediction estimation. Repeating this procedure 5 times provided us with 400 predictions that we could compare to the true categories. By running a permutation test 100000 times on electrodes with randomly permuted class labels we estimated that 99.999th percentile (equivalent to significance threshold of $p\le 0.00001$) of *F*_*1*_ *score* is 0.390278. F_1_ score is an aggregated metric of the performance of a classifier that combines both the *precision* (the ratio of the data samples that truly belong to a category among the ones that were assigned to that category by the model) and *recall* (the ratio of data samples that were correctly identified to belong to a category to the total number of samples of that category in the dataset) into one number: ${{\rm{F}}}_{1}=2\cdot \frac{{\rm{precision}}\cdot {\rm{recall}}}{{\rm{precision}}+{\rm{recall}}}$. In total 787 electrodes had a predictive power of F_1_ > 0.390278 in at least one of the categories. Although none of the results of this work depend on direct comparison of F_1_ scores, we have estimated the noise level of this metric by computing standard deviation over 5 folds of each electrode and calculated the average standard deviation of F_1_ metric to be $\sigma =0.1008$ over all predictive electrodes. For each of those electrodes a Random Forest model was retrained once more on whole data (400 samples instead of 320) and that model was used for calculating feature importances and, ultimately, for understanding which parts of the recorded activity were relevant for visual object recognition in human brain.

### Feature importance for the analysis of task-relevant neural activity

During the process of constructing the decision trees, Random Forest relies on some features more than on the others. We chose *Gini impurity*^89^ as a measure of which features should be used to make the branching decisions in the nodes of a tree. This score, along with the number of times each particular feature was used across trees, informed us on the relative importance of each particular feature with respect to other features. Gini impurity $G$ is calculated as

1 $$G=\mathop{\sum }\limits_{i=1}^{i={n}_{c}}\,{p}_{i}(1-{p}_{i}),$$

where *n*_*c*_ is the number of categories and *p*_*i*_ is the proportion of class *i* in a node. To pick a feature for a parent node, Gini impurity of both child nodes of that parent are calculated and used to estimate the *reduction in impurity* that will be achieved by picking that particular features as the branching factor for the node. The feature that decreases impurity the most is selected to be the branching factor of that parent node. The reduction in impurity is calculated as

2 $$I={G}_{{\rm{parent}}}-{G}_{{\rm{left}}{\rm{child}}}-{G}_{{\rm{right}}{\rm{child}}}$$

and is called *node importance*. *Feature importance* of a feature *f* is estimated by calculating the sum of Gini impurity reductions over all samples in the dataset that were achieved with the use of a particular feature *f* and normalizing it by the total number of samples. Figure 1e is a visual representation of relative feature importance, color intensity shows the importance of each of 7008 (146 frequencies × 48 time bins) spectrotemporal features from one probe. In total our analysis has produced 787 × 8 such images – one for each probe-class pair.

The importance map computed as depicted on Fig. 1 is a global map for all 8 categories, such that the regions that are highlighted on the map are important for distinguishing between all 8 categories. There is, however, a way to look at category-specific importances as well. The final set of nodes of a decision tree, called *leaves*, are the end-points of the classification process and each leaf is associated with a certain category. If we take one TF activity map (TF components are the features) and start traversing a decision tree following the rules set by the nodes of the tree, we will end up in a certain leaf. That leaf will be associated with a certain category, for example, with faces. The fact that we followed the rules and ended up in that leaf indicates that the TF map we used as the input to the tree probably comes from a trial where a face stimulus was shown to the subject. In order to get category-specific feature importance map we took all the leaves associated with a category, traversed the tree backwards and tracked all the features that were used on the path from the leaf to the root of the tree. This way we got a list of features (TF components) that were used to identify a neural response as belonging to a certain category. Random Forest feature importance allowed us to identify which sub-regions of neural activity (TF maps) are relevant for decoding. It showed that only a small portion of activity is actually crucial for identifying the categories.

To compare importance maps between each other we fit a normal distribution on the difference between two maps and considered statistically significant the differences that are bigger than *μ* + 4*σ*. One spectrotemporal importance map consists of 7008 values. To filter out false positives we stipulated that only 1 false positive out of 7008 pixels can be tolerated and tuned the threshold accordingly. That requirement resulted in the p-value of 0.0001427 and confidence level of 99.99%, corresponding to 3.89*σ*, which we rounded up to $\sigma =4.0$.

### Hierarchical clustering to reveal types of activity patterns

To further analyze the spectrotemporal signatures elicited by different visual categories in different parts of human brain we clustered filtered activity patterns and identified the most prominent groups. The result of this analysis is shown in the second column of Fig. 6. For each category, the four most populated (in terms of the number of probes) clusters of activity patterns elicited by this category are shown.

**Figure 8:**
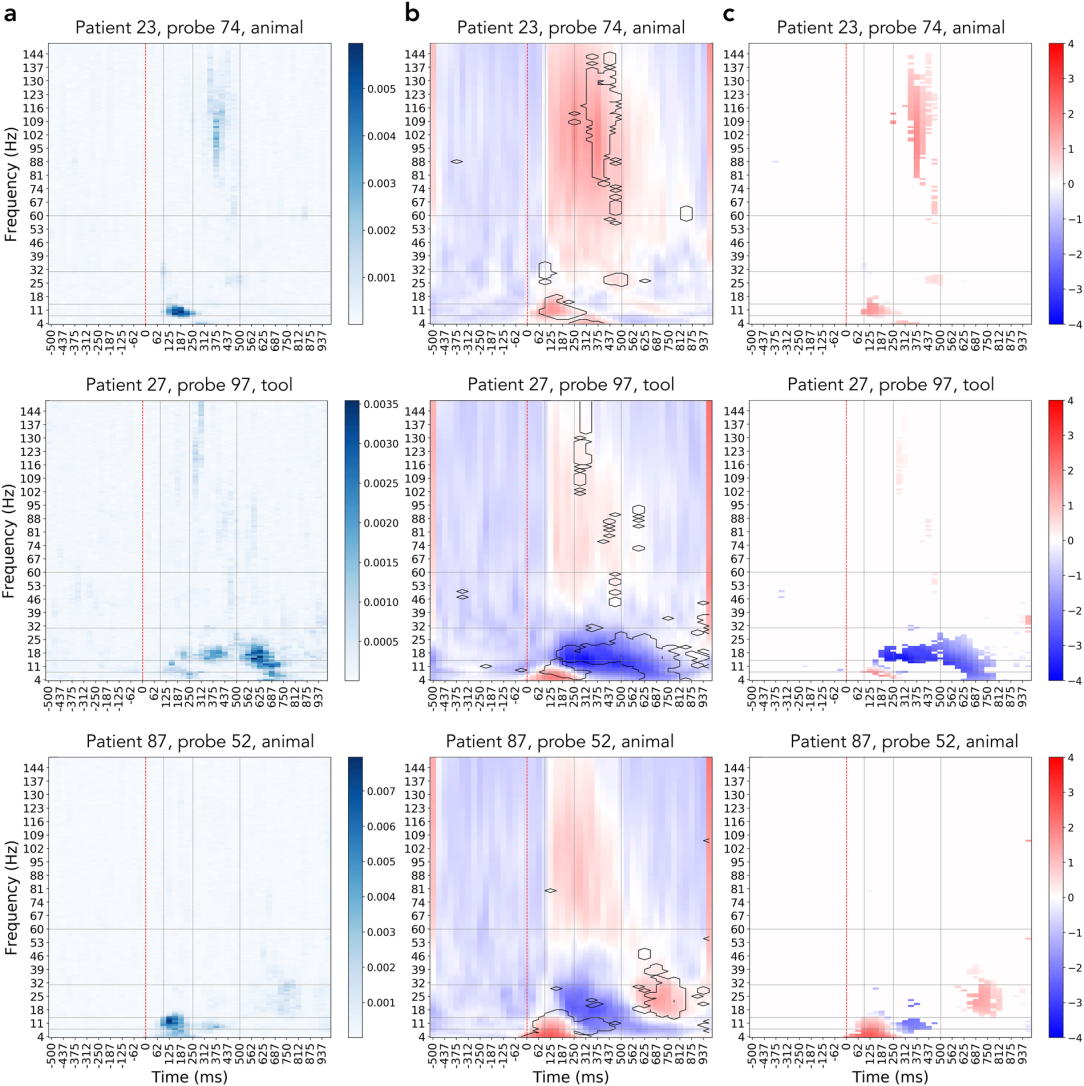
Using the importance map to filter out irrelevant activity. The three rows show three different examples of how filtering of the activity by importance is beneficial: in patient 23, probe 74 we see that only later portion of the broadband gamma activity increase was useful for identifying this activity as a response to the animal stimulus; patient 27, probe 97 shows that although there is an increase in broadband activity, the actually useful information contained in decrease in the lower frequency bands; patient 87, probe 52 demonstrates that for decoding this particular probe’s activity one must focus on the activity in lower frequencies at specific time and, despite prominent presence, ignore the increase in broadband gamma. (**a**) Probe’s importance map, color codes the relative importance of each spectrotemporal feature within the map. (**b**) Full spectrotemporal activity of the probe, features with importances one standard deviation higher than the average (in contour) mark the regions of activity that were useful for the decoding model. Color shows the activity in the power spectrum normalized by the baseline. (**c**) Activity of the probes filtered by the importance mask, only the relevant activity is preserved. Color shows the activity in the power spectrum normalized by the baseline.

To do the clustering we first took each probe’s category-specific activity separately by averaging probe’s responses to 50 images of each particular category in time-frequency domain. We then masked the activity with the category importance map (as shown on Fig. 8), leaving only those features out of 146 × 48 that have importance score larger that *μ* + *σ*, where *μ* is the average importance score for that category and *σ* is one standard deviation of the score distribution.

Masked activity patterns were hierarchically clustered using Eq. 3 to calculate the distance between a pair of clusters *U* and *V* as the maximal cosine distance between all of the clusters’ member observations (complete linkage clustering):

3 $$d(U,V)=\,{\max }\left(\frac{{\bf{u}}\cdot {\bf{v}}}{\parallel {\bf{u}}\parallel \,\parallel {\bf{v}}\parallel }\right)\,\forall {\bf{u}}\in U,\,\forall {\bf{v}}\in V$$

SciPy^90^ implementation of the hierarchical clustering methods was used in this work. Resulting clustering assignments were visually inspected and corrected.

## Supplementary information

Supplementary information.

## Data Availability

The final pre-processed spectrotemporal data are available for download under Academic Free License 3.0 from https://web.gin.g-node.org/ilyakuzovkin/Spectral-Signatures-of-Perceptual-Categorization-in -Human-Cortex. The code that was used to produce this data from the raw recordings and to perform all of the subsequent analysis steps is available at https://github.com/kuz/Spectral-signatures-of-perceptual-categori zation-in-human-cortex. All raw human brain recordings that support the findings of this study are available from Lyon Neuroscience Research Center but restrictions apply to the availability of these data, which were used under license for the current study, and are not publicly available. Raw data are however available from the authors upon reasonable request and with permission of Lyon Neuroscience Research Center.
