# Supplementary material for: Identifying task-relevant spectral signatures of perceptual categorization in the human cortex
Source: Sci Rep. 2020 May 12;10:7870. doi: 10.1038/s41598-020-64243-6 (PMC7217881; doi:10.1038/s41598-020-64243-6)
Supplement: Supplementary file 1 — Supplementary information. [file 41598_2020_64243_MOESM1_ESM.pdf]

Supplementary information to the research article "Identifying task-relevant spectral signatures of perceptual categorization in the human cortex"

Ilya Kuzovkin[1,*], Juan R. Vidal[2,6,*,+], Marcela Perrone-Bertolotti[3], Philippe Kahane[4,5], Sylvain Rheims[6,7], Jaan Aru[1,8], Jean-Philippe Lachaux[6,9], and Raul Vicente[1,*,+]

[1]Computational Neuroscience Lab, Institute of Computer Science, University of Tartu, Tartu, Estonia
[2]UMRS 449, Université Catholique de Lyon / Ecole Pratique des Hautes Etudes 10 Place des Archives 69002 Lyon, France.
[3]University Grenoble Alpes, University Savoie Mont Blanc, CNRS, LPNC, 38000 Grenoble, France
[4]Inserm, U1216, F-38000 Grenoble, France
[5]Neurology Department, CHU de Grenoble, Hôpital Michallon, F-38000 Grenoble, France
[6]INSERM U1028, CNRS UMR5292, Lyon Neuroscience Research Center, Lyon, France
[7]Department of Functional Neurology and Epileptology, Hospices Civils de Lyon and Université Lyon, Lyon, France
[8]Institute of Biology, Humboldt University Berlin, Germany
[9]Université Claude Bernard, Lyon, France
[*]ilya.kuzovkin@gmail.com, juanrvidal@gmail.com, raulvicente@gmail.com
[+]these authors contributed equally to this work

Figures

All supplementary figures are available in an online public `figshare.com` repository[1]. The repository contains the following items:

(a) figures similar to Figure 2a of the paper, showing that only a portion of responsive probes is predictive,
DOI: `https://doi.org/10.6084/m9.figshare.11413965.v1`,

(b) all figures showing the difference between polypredictive and monopredictive neural locations,
DOI: `https://doi.org/10.6084/m9.figshare.8223398.v1`,

(c) all figures of normalized TF activity with importance contour overlay,
DOI: `https://doi.org/10.6084/m9.figshare.8223389.v1`,

(d) all clustering figures of important activity into spectral signatures,
DOI: `https://doi.org/10.6084/m9.figshare.8223383.v1` and

(e) all time-frequency importance maps, organized into average, per-subject, and per-area groups,
DOI: `https://doi.org/10.6084/m9.figshare.8223356.v1`.

The original stimuli images that were used in the experiment are available from the authors upon request.

[1]https://figshare.com/projects/Supplementary$_{F}igures_{f}or_{I}dentifying_{t}ask-relevant_{s}pectral_{s}ignatures_{o}f_{p}erceptual_{c}ategorization_{i}n_{t}he_{h}uman_{c}ortex_{/}$64523

Tables

BA	Area[1]	house	face	anim	scene	tool	pseud	chars	scram	All
0	Out of BA atlas	1_{232}	6_{228}	8_{244}	4_{227}	3_{234}	25_{269}	6_{208}	6_{240}	59_{1882}
1	Primary somatosensory	0_{2}	0_{2}	0_{2}	0_{2}	0_{2}	0_{1}	0_{2}	0_{2}	0_{15}
2	Primary somatosensory	0_{16}	0_{16}	0_{19}	0_{17}	0_{17}	1_{17}	0_{16}	0_{17}	1_{135}
3	Primary somatosensory	0_{24}	0_{21}	0_{22}	0_{24}	0_{17}	0_{23}	0_{18}	0_{21}	0_{170}
4	Primary motor	0_{18}	0_{16}	0_{15}	0_{16}	0_{16}	0_{15}	0_{14}	0_{16}	0_{126}
5	Somatosensory association	0_{5}	0_{3}	0_{4}	0_{4}	0_{3}	0_{4}	0_{4}	0_{5}	0_{32}
6	Premotor cortex	0_{47}	0_{47}	5_{42}	0_{43}	0_{42}	2_{54}	0_{41}	0_{42}	7_{358}
7	Secondary sensorimotor	0_{10}	0_{4}	1_{6}	0_{6}	3_{11}	0_{10}	0_{4}	0_{3}	4_{54}
8	Frontal eye fields	0_{5}	0_{5}	0_{6}	0_{7}	0_{9}	0_{8}	0_{6}	0_{6}	0_{52}
9	Dorsolateral prefrontal	0_{15}	0_{17}	1_{17}	0_{15}	0_{15}	0_{15}	0_{14}	0_{13}	1_{121}
10	Anterior prefrontal	0_{12}	0_{9}	0_{9}	0_{10}	0_{13}	0_{15}	0_{10}	0_{12}	0_{90}
11	Orbitofrontal area	0_{42}	2_{41}	2_{47}	0_{41}	0_{45}	1_{50}	1_{36}	2_{40}	8_{342}
12	Orbitofrontal area	0_{0}	0_{0}	0_{0}	0_{0}	0_{0}	0_{0}	0_{0}	0_{0}	0_{0}
13	Insular cortex	0_{0}	0_{0}	0_{0}	0_{0}	0_{0}	0_{0}	0_{0}	0_{0}	0_{0}
14		0_{0}	0_{0}	0_{0}	0_{0}	0_{0}	0_{0}	0_{0}	0_{0}	0_{0}
15		0_{0}	0_{0}	0_{0}	0_{0}	0_{0}	0_{0}	0_{0}	0_{0}	0_{0}
16	Insular cortex	0_{0}	0_{0}	0_{0}	0_{0}	0_{0}	0_{0}	0_{0}	0_{0}	0_{0}
17	Primary visual (V1)	0_{12}	1_{9}	0_{11}	1_{14}	0_{9}	3_{9}	2_{7}	9_{14}	16_{85}
18	Secondary visual (V2)	9_{41}	10_{33}	4_{36}	1_{38}	3_{30}	10_{31}	3_{27}	19_{33}	59_{269}
19	Inferior occipital (V3, V4)	9_{92}	29_{76}	35_{96}	13_{84}	17_{90}	15_{73}	17_{52}	28_{71}	163_{634}
20	Inferior temporal	1_{195}	24_{209}	11_{186}	1_{203}	12_{198}	12_{238}	1_{176}	1_{205}	63_{1610}
21	Middle temporal	0_{78}	5_{77}	1_{84}	0_{84}	0_{86}	5_{89}	0_{74}	0_{80}	11_{652}
22	Superior temp., Wernicke's	0_{36}	0_{33}	0_{32}	0_{36}	0_{35}	8_{36}	0_{32}	0_{34}	8_{274}
23	Cingulate cortex	0_{19}	0_{22}	0_{20}	0_{22}	0_{22}	0_{21}	0_{23}	0_{20}	0_{169}
24	Cingulate cortex	0_{5}	0_{5}	1_{6}	0_{7}	0_{7}	0_{6}	0_{4}	0_{7}	1_{47}
25	Subgenual area	0_{0}	0_{0}	0_{0}	0_{0}	0_{0}	0_{0}	0_{0}	0_{0}	0_{0}
26	Retrosplenial region	0_{2}	0_{3}	0_{3}	0_{3}	0_{3}	0_{4}	0_{2}	0_{3}	0_{23}
27	Piriform cortex	0_{2}	0_{3}	0_{1}	0_{3}	1_{2}	0_{2}	0_{4}	0_{2}	1_{19}
28	Cingulate cortex	0_{4}	2_{3}	0_{2}	0_{5}	0_{2}	0_{2}	0_{2}	0_{3}	2_{23}
29		0_{0}	0_{0}	0_{0}	0_{0}	0_{0}	0_{0}	0_{0}	0_{0}	0_{0}
30	Posterior cingulate	1_{3}	3_{4}	2_{6}	0_{5}	3_{6}	3_{7}	0_{1}	2_{4}	14_{36}
31		0_{0}	0_{0}	0_{0}	0_{0}	0_{0}	0_{0}	0_{0}	0_{0}	0_{0}
32		0_{21}	0_{23}	1_{26}	0_{20}	0_{21}	0_{25}	0_{22}	0_{24}	1_{182}
33	Cingulate cortex	0_{0}	0_{0}	0_{0}	0_{0}	0_{0}	0_{0}	0_{0}	0_{0}	0_{0}
34	Dorsal entorhinal	0_{10}	3_{9}	0_{9}	0_{9}	0_{10}	0_{10}	0_{10}	0_{9}	3_{76}
35	Perirhinal, ectorhinal	0_{8}	0_{4}	0_{5}	0_{5}	0_{6}	0_{6}	0_{4}	0_{6}	0_{44}
36	Perirhinal, ectorhinal	0_{19}	5_{19}	4_{24}	0_{22}	4_{24}	1_{20}	0_{17}	0_{19}	14_{164}
37	Fusiform gyrus	25_{157}	55_{165}	43_{174}	22_{163}	31_{179}	55_{157}	30_{117}	46_{155}	307_{1267}
38	Temporal pole	0_{15}	1_{16}	0_{14}	0_{12}	0_{18}	0_{16}	0_{10}	0_{15}	1_{116}
39	Angular gyrus	0_{16}	2_{15}	2_{19}	0_{15}	0_{16}	2_{16}	1_{14}	2_{15}	9_{126}
40	Supramarginal gyrus	0_{26}	0_{23}	0_{25}	0_{27}	0_{29}	2_{25}	0_{24}	0_{27}	2_{206}
41	Primary auditory	0_{10}	0_{11}	0_{12}	0_{12}	0_{11}	0_{13}	0_{10}	0_{10}	0_{89}
42	Primary auditory	0_{7}	0_{7}	0_{7}	0_{7}	0_{7}	2_{7}	0_{7}	0_{7}	2_{56}
43	Primary gustatory	0_{4}	0_{3}	0_{4}	0_{4}	0_{4}	0_{4}	0_{3}	0_{4}	0_{30}
44	Broca's area	0_{3}	0_{3}	0_{5}	0_{3}	1_{4}	0_{4}	0_{3}	0_{4}	1_{29}
45	Broca's area	0_{12}	0_{11}	2_{13}	0_{11}	0_{11}	1_{12}	0_{10}	1_{11}	4_{91}

46	Dorsolateral prefrontal	0 17	0 20	1 21	0 15	0 19	0 19	0 16	0 19	1 146
47	Inferior frontal	0 25	2 23	0 28	0 23	0 25	0 27	0 24	0 21	2 196
48	Retrosubicular area	0 188	2 190	6 186	0 182	0 185	14 224	0 175	0 186	22 1516
	All	46 1455	152 1428	130 1488	42 1446	78 1483	162 1584	61 1243	116 1425	787 11552

Table 1. Number of responsive and predictive probes across categories (columns) and Brodmann areas (rows). Format “Predictive responsive" shows the number of predictive probes in a large font and the number of responsive probes in a small font.

References

1. BELL, D. & Gaillard, F. Brodmann areas (2018).
